# The efficacy of olfactory training in improving olfactory function: a meta-analysis

**DOI:** 10.1007/s00405-024-08733-7

**Published:** 2024-05-27

**Authors:** Alice Helena Delgado‑Lima, Jaime Bouhaben, María Luisa Delgado‑Losada

**Affiliations:** Experimental Psychology, Cognitive Processes and Speech Therapy Department, Faculty of Psychology, Complutense University of Madrid, 28223 Pozuelo de Alarcón, Spain

**Keywords:** Olfactory function, Olfactory dysfunction, Olfactory training, Therapeutic approach

## Abstract

**Purpose:**

Study the efficacy of olfactory training in smell recovery.

**Methods:**

An extensive search was performed through different databases in order to find articles analyzing the efficacy of olfactory training as a treatment for olfactory dysfunction. Methodological quality of primary studies within the final sample was assessed following PRISMA guidelines. Standardized mean differences in pre–post olfactory training groups, and also in experimental-control and pre-follow up if possible, were computed by Hedges’ g effect size statistic. Each effect size was weighted by its inverse variance.

**Results:**

Final sample was composed of 36 articles (45 pre–post effect sizes). Contrasts were performed separately for odor identification, odor discrimination, odor threshold and general olfactory function. Moderate to large and heterogeneous effect was obtained for olfactory function (g = 0.755, k = 45, SE = 0.093, CI 95% = [0.572, 0.937]), different moderators had a significant effects, such as, training duration, age and anosmia diagnosis.

**Conclusion:**

Olfactory training was found to have a positive and significant effect on rehabilitating the olfactory function.

## Introduction

Olfactory dysfunctions (OD) are a problem to which less attention is usually paid because it is considered to have a less serious clinical impact, because olfactory dysfunctions are rarely fatal, patients often do not receive adequate medical care. An impaired sense of smell can have a negative effect on quality of life and safety, and can be a sign of other health problems [1–3].

Recent investigations have shown that OD occurs at a much higher rate than previously assumed. The frequency of a decreased olfactory performance was estimated as high as 16%, with approximately 5% of the general population being functionally anosmic [4]. In adults aged 50 years and above, the prevalence of impaired olfaction was found to be 25%, implicating that aging is an important factor associated with olfactory loss [5, 6]. In addition, sinonasal disease, upper respiratory tract infections, and trauma are among the most frequent causes of dysosmia [7].

In patients with OD, an impact is usually observed on activities of daily living, personal hygiene, safety and sexual behavior [8, 9]. It has been suggested that the malnutrition associated with age is due OD alone or associated with taste alteration derived by retronasal olfactory alterations [10].

Currently, there is no generally approved way to treat olfactory impairment, however, the neural plasticity quality of the olfactory system offers possibilities regarding treatment. Regenerative capacities of the olfactory pathway include mechanisms ranging from changes in membrane excitability to changes in synaptic efficacy to neurogenesis and apoptosis [11]. More importantly, it has been shown that exposure to an odor may modulate this regenerative capacity [12, 13]. As odors can influence this regenerative capacity, olfactory training (OT) has been a basis of research conducted in various smell and taste studies [14].

In the absence of effective pharmacological treatment [15–18]. OT has emerged as a first tool for the treatment of olfactory loss. The concept of training is analogous to physical therapy following a cerebrovascular accident, a stroke or other neurological condition. In the face of an event that generates a deficit, existing neurological pathways can be strengthened and trained to compensate [19]. Olfactory training is based on retraining the brain to correctly interpret the neurological signals received when odorants are presented and generate an impulse that travels through the olfactory nerve, the olfactory bulb and the olfactory cortex. Olfactory training involves the deliberate and repeated exposure of a set of odorants over a period of time. Studies have demonstrated its efficacy in post-infection patients on rehabilitating olfactory function [20, 21].

Two modalities of olfactory training have been described: classic olfactory training where patients are exposed to the same 4 odorants (rose, eucalyptus, lemon, and clove) during the rehabilitation process, and personalized or modified olfactory training, adapted to the patient’s results in the tests measuring quantity and quality of olfactory loss or based on different concentrations [22], and other olfactory training modalities were also included such as, aromatherapy [23] or essential mineral oils [24], where patients are exposed to different groups of odorants that are modified periodically throughout the intervention, and must be smelled for at least 5 min, 2 times a day and for at least 6 weeks [25–27].

Nonetheless, given that there are open discussions about the effectiveness of OT and that not under all circumstances the training proved to lead to significant smell improvement (e.g. in elderly) [28, 29].

In view of the open discussions about olfactory training effectiveness, this study was conducted with the view to summarize, update and obtain reliable and precise estimates of overall training benefit on the olfactory function, and to analyze the heterogeneity between the available studies to date before its wide application in clinical practice.

## Methods and materials

### Search strategy

An extensive literature search and review was carried out to identify studies that conducted an olfactory evaluation before and after olfactory training. Articles whose publication period was between 2010 and March, 2022 were taken into account.

Initially, articles were searched in English, Spanish and Portuguese. Electronic database retrieval included ProQuest, Pubmed, Scopus, Web of science using the following keywords: “olfactory”, “smell”, “smelling”, “odor”, “sniffin”, “olfaction”, “anosmia”, “olfactory dysfunction”, “olfactory function”, “olfactory performance” combined with “treatment”, “stimulation”, “trial”, “therapy”, “intervention”, “olfactory treatment”, “training” and “rehabilitation”. Keywords were adapted for each database. Moreover, references of all studies were also examined for possible additional publications. Later on, another search was yielded using Google Academic in order to retrieve possible additional studies that may have been left over on the initial search.

### Review strategy

According to the research topic and summary, two researchers independently (AHDL and MLDL) identified possible valid studies based on title and abstract. Following, a pair of raters selected abstracts for full review based on inclusion/exclusion criteria. Two of the authors (AHDL and JB), using a standardized form, extracted data, narrative synthesis and vote counting methods were used to summarize and interpret study data. Any disagreement was resolved by consulting a third party investigator (MLDL). The current study was reported in accordance with the latest PRISMA guidelines [30].

### Quality assessment

Qualitative assessment of the risk of bias for each primary study included within the final sample was performed. The Risk Of Bias In Non-randomized Studies of Interventions (ROBINS-I) [31] tool was chosen in order to accomplish this quality assessment. The ROBINS-I is an instrument from the Cochrane Collaboration whose main function is to assess non-randomized studies of interventions. Overall risk-of-bias is judged under six potential sources of bias: confounding bias risk, Selection bias risk, bias risk due to classification of interventions, bias risk due to deviation from intended intervention, measurement bias risk and reporting bias risk. Within each domain or source of potential bias, assessments are made for one or more items, which may cover different aspects of the domain, or different outcomes.

Potential risk-of-bias for each source/domain may range from Low to Moderate to Serious to Critical [or NI (not informed), if that would be the case]. Two researchers (AHDL and JB) performed the assessment and discussed all differences in order to establish an agreement for each study’s risk-of-bias.

### Eligibility criteria

The primary outcome of this study is olfactory training. All papers selected for final consideration met the following criteria: (i) participants signed informed study consent; (ii) participants were submitted to an olfactory evaluation test previous to training; (iii) participants were submitted to a otorhinolaryngology exploration previous to training; (iv) the treatment provided was non-pharmacological olfactory training or a combination of both; (v) participants were provided an olfactory training kit or the means to stimulate olfactory function; (vi) training consisted of a minimum of 6 weeks; (vii) participants were evaluated after finalizing the training period; (viii) results of olfactory training were reported.

### Coding procedures

Two of the authors (AHDL and JB) independently coded each article for relevant information including sample selection, sample size, main statistics necessary to the computation of effect size, and information necessary for the moderator analyses.

### Statistical methods

The complete set of analyses was performed with the metafor package [32] of R software, version 3.5.2 [33]. All analyses were performed with significance level set at α = 0.05.

Firstly, effect sizes were calculated as Hedges’ g (standardized mean differences, SMD). Three different effect sizes were computed for each study: pre–post (SMD between pre and post olfactory scores in training groups), experimental-control (SMD between post scores of training and control groups) and pre-follow up (SMD between pre and follow-up olfactory scores in training groups). For some studies, mean and standard deviations were not available, so F tests or t-statistics were used to calculate effect size. Differences between study sample sizes were controlled by weighting each study according to its inverse variance estimate. This procedure was formulated by Hedges [34].

Once Hedges’ g and variance estimates were computed for each primary study, random-effect models were estimated in order to obtain meta-analytic effect sizes. Meta-analytic effect size was calculated for olfactory identification, olfactory discrimination, olfactory threshold and olfactory function (TDI) in pre–post, experimental-control and pre-follow up categories. Furthermore, Cochran’s Q tests were performed for each model to study effect size homogeneity across primary studies. In addition, publication bias was addressed by four different approaches. Firstly, evidence of publication bias was searched through visual inspection of funnel plots. Then, Egger’s regression test [35] was performed in order to assess potential asymmetry of those funnel plots. Finally, Duval and Tweedie’s trim and fill [36] method was applied, alongside with the computation of fail-safe N number (number of null studies which are required to be introduced into the meta-analysis to cancel the observed effect) by Rosenthal [37] method.

### Moderators

As statistically significant heterogeneity within meta-analytic models is expected, key moderators of interest were coded for each study. Moderator analysis comprised the following variables: (i) olfactory diagnosis (categorical; hyposmia, anosmia or others (diagnosis was obtain in each study included, when it was not provided in the study, it was obtained by the TDI 10th cut-off point to discriminate between hyposmia and anosmia, as established in the original study by Hummel and colleagues [38]), (ii) etiology of olfactory loss (categorical; otorhinolaryngological issues, respiratory infection, head trauma, laryngeal tumor, idiopathic alterations, persistent COVID-19 or healthy individuals), (iii) type of treatment (categorical; only olfactory training, olfactory training combined with prescribed medication or others), (iv) olfactory training type (categorical; classic training, personalized training or other), (v) age (numeric, mean age of participants), and (vi) training duration in weeks (numeric).

In addition, in experimental vs control analysis, olfactory diagnosis of control groups (categorical; hyposmia, anosmia, others or healthy individuals) was also included as moderator. In pre vs follow-up analysis, weeks between pre and follow-up assessments were included.

## Results

### Search results

**Fig. 1:**
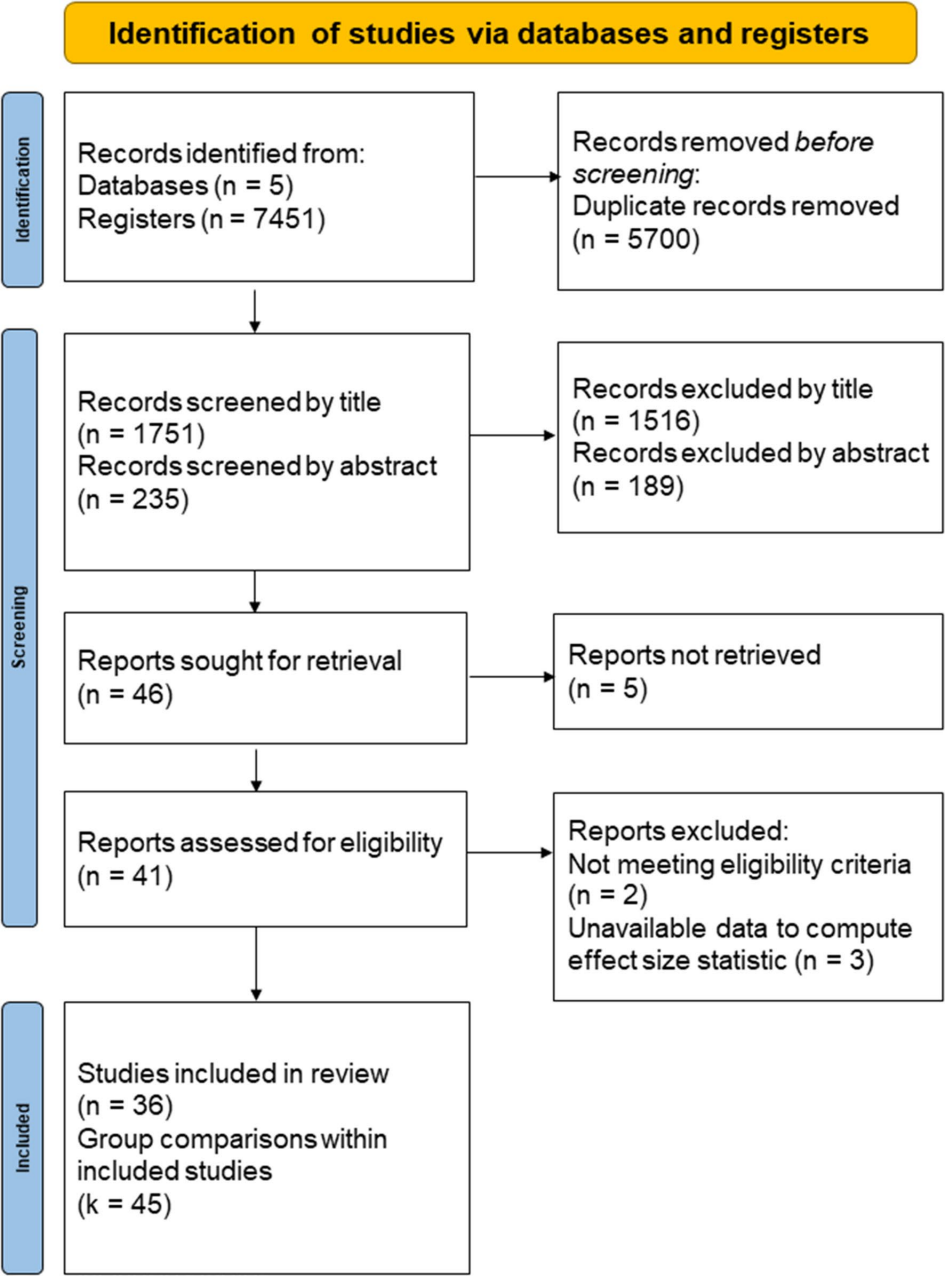
PRISMA flow diagram of primary studies

Overall, 7451 articles were found in the initial search. The relation between databases and articles is as follows: Scopus (3299 titles), ProQuest (2761 titles), Pubmed (1206 titles) and Web of Science (185 titles). Additionally, a thorough manual review of articles was performed using cross-references from identified original articles and reviews (2 titles). Before any other action, 5700 duplicates were removed. The remaining 1751 references were screened, first, by title (1516 excluded) and then by abstract (189 excluded). Finally, 46 references were intended to be retrieved and 36 studies were eventually included in the final sample (45 pre–post comparisons). Search results are summarized in PRISMA Flow Diagram (Fig. 1).

A brief summary of these 36 studies is exposed in Table 1. Each study is characterized with Author, Year and Nationality. Methodological variables are the olfactory assessment instrument, olfactory scores obtained with this instrument, presence of a control group and a brief description of the olfactory intervention carried out. Results of quality assessment with Cochrane’s ROBINS-I tool are exposed in Table 2.

**Table 1:** Olfactory training studies included in meta-analysis

First author	Year	Country	Olfactory instrument	Olfactory test scores	Intervention	Control group	Ne	Nc
Abdelhafeez et al. [39]	2022	Egypt	Sniffin’ sticks	TDI, T, D, I	Classic training	N	196	–
Altundag et al. (i) [29]	2015	Turkey	Sniffin’ sticks	TDI, T, D, I	Personalized training	Y	37	15
Altundag et al. (ii) [29]	2015	Turkey	Sniffin’ sticks	TDI, T, D, I	Classic training	Y	33	15
Birte-Antina et al. [40]	2017	Germany	Sniffin’ sticks	TDI, T, D, I	Classic training	Y	60	31
Bratt et al. [41]	2020	Norway	Sniffin’ sticks	TDI, T, D, I	Classic training and oral corticosteroids	N	22	–
Damm et al. (i) [42]	2014	USA	Sniffin’ sticks	TDI, T, D, I	High concentrations classic training	N	70	–
Damm et al. (ii) [42]	2014	USA	Sniffin’ sticks	TDI, T, D, I	Low concentrations classic training	N	74	–
Denis et al. [43]	2021	France	VAS scale	TDI	Classic training	N	548	–
Fleiner et al. (i) [44]	2012	Germany	Sniffin’ sticks	TDI, T, D, I	Personalized training	N	28	–
Fleiner et al. (ii) [45]	2012	Germany	Sniffin’ sticks	TDI, T, D, I	Personalized training and Steroids	N	18	–
Geißler et al. [45]	2013	Germany	Sniffin’ sticks	TDI, T, D, I	Classic training	N	39	–
Gellrich et al. [46]	2017	Germany	Sniffin’ sticks	TDI, T, D, I	Classic training	Y	30	31
Genetzaki et al. (i) [47]	2021	Greece	Sniffin’ sticks	TDI	Classic training and oral methylprednisolone	N	78	–
Genetzaki et al. (ii) [47]	2021	Greece	Sniffin’ sticks	TDI	Classic training	N	53	–
Gurbuz et al. [48]	2021	Brazil	CCRC	TDI	Classic training	N	11	–
Haehner et al. [49]	2013	Germany	Sniffin’ sticks	TDI, T, D, I	Classic training	Y	35	35
Hosseini et al. [50]	2020	Iran	Sniffin’ sticks	TDI, T, D, I	Classic training	Y	8	11
Jiang et al. (i) [51]	2019	Taiwan	UPSIT	I	Classic training	N	45	–
Jiang et al. (ii) [51]	2019	Taiwan	UPSIT	I	Phenyl ethyl alcohol	N	45	–
Jiang et al. (i) [24]	2017	Taiwan	UPSIT	I	Phenyl ethyl alcohol	N	42	–
Jiang et al. (ii) [24]	2017	Taiwan	UPSIT	I	Mineral oil	N	39	–
Jiramongkolchai et al. [52]	2021	EUA	Sniffin’ sticks	TDI, T, D, I	Classic training and budesonide nasal irrigation	N	16	–
Knudsen et al. (i) [53]	2015	Denmark	Sniffin’ sticks	D, I	Classic training and anti-parkinsonian medication	Y	34	20
Knudsen et al. (ii) [53]	2015	Denmark	Sniffin’ sticks	D, I	Classic training and anti-parkinsonian medication	Y	34	26
Kollndorfer et al. [54]	2014	Austria	Sniffin’ sticks	TDI, T, D, I	Personalized training	N	11	–
Konstantinidis et al. (i) [55]	2016	Greece	Sniffin’ sticks	TDI, T, D, I	Classic training	Y	36	41
Konstantinidis et al. (ii) [55]	2016	Greece	Sniffin’ sticks	TDI, T, D, I	Classic training	Y	34	41
Konstantinidis et al. (i) [26]	2013	Greece	Sniffin’ sticks	TDI, T, D, I	Classic training	Y	49	32
Konstantinidis et al. (ii) [26]	2013	Greece	Sniffin’ sticks	TDI, T, D, I	Classic training	Y	23	15
Lamira et al. [56]	2019	USA	Sniffin’ sticks	TDI, T, D, I	Classic training	N	18	–
Langdon et al. [57]	2018	Spain	BAST-24	D, R, I	BASTAT-6	Y	21	21
Le Bon et al. (i) [58]	2021	Germany	Sniffin’ sticks	TDI	Classic training and oral corticosteroids	N	9	–
Le Bon et al. (ii) [58]	2021	Germany	Sniffin’ sticks	TDI	Classic training	N	18	–
Liu et al. [59]	2021	Germany	Sniffin’ sticks	TDI	Classic training	N	246	–
Mahmut et al. (i) [60]	2021	Germany	Sniffin’ sticks	TDI, T, D, I	Nasal clip	N	25	–
Mahmut et al. (ii) [60]	2021	Germany	Sniffin’ sticks	TDI, T, D, I	Nasal clip	N	25	–
Mahmut et al. (iii) [61]	2020	Germany	Sniffin’ sticks	TDI, T, D, I	Classic training	Y	27	27
Morquecho-Campos et al. (i) [62]	2019	Sweeden	None	I	Personalized training	N	13	–
Morquecho-Campos et al. (ii) [62]	2019	Sweeden	None	I	Personalized training	N	14	–
Negoias et al. [63]	2016	EUA	Sniffin’ sticks	T and I	Classic training	N	97	–
Oleszkiewicz et al. (i) [64]	2018	Germany	Sniffin’ sticks	TDI, T, D, I	Classic, personalized and odor mixtures	N	57	–
Oleszkiewicz et al. (ii) [64]	2018	Germany	Sniffin’ sticks	TDI, T, D, I	Classic, personalized and odor mixtures	N	51	–
Oleszkiewicz et al. (i) [23]	2022	Germany	Sniffin’ sticks	T, D, I	Single molecule odor	Y	32	29
Oleszkiewicz et al. (ii) [23]	2022	Germany	Sniffin’ sticks	T, D, I	Odor mixtures	Y	23	29
Pellegrino et al. (i) [27]	2019	USA	Sniffin’ sticks	T and I	Classic training	N	24	–
Pellegrino et al. (ii) [27]	2019	USA	Sniffin’ sticks	T and I	Classic training	N	18	–
Qiao et al. [65]	2019	China	Sniffin’ sticks	TDI, T, D, I	Personalized training	Y	65	60
Rezaeyan et al. (i) [22]	2022	Iran	Sniffin’ sticks	TDI, T, D, I	Classic training	Y	9	9
Rezaeyan et al. (ii) [22]	2022	Iran	Sniffin’ sticks	TDI, T, D, I	Personalized training	Y	7	9
Saatci et al. (i) [25]	2020	Germany	Sniffin’ sticks	TDI, T, D, I	Classic training	N	30	–
Saatci et al. (ii) [25]	2020	Germany	Sniffin’ sticks	TDI, T, D, I	Olfactory training ball	N	30	–
Yan et al. [66]	2018	China	Sniffin’ sticks	TDI	Classic training	N	80	–
Yoon Choi et al. [67]	2021	Korea	KVSS	TDI, T, D, I	Personalized training	Y	40	64

Regarding olfactory test scores, T accounts for Threshold score, D for discrimination score and I for Identification score, whereas TDI represents overall olfactory score. VAS scale goes for olfactory self-assessment through visual analog scale

In control group, Y (yes) or N (no) refers to the presence of a control group within the study

Ne goes for sample size in experimental group and NC for sample size in control group, if exists

**Table 2:** Quality assessment for each primary study with Cochrane’s risk-of-bias tool deviation

First author	Year	Confounding bias risk	Selection bias risk	Classification of interventions	from intended intervention bias risk	Missing data bias risk	Measurement bias risk	Reporting bias risk	Overall bias risk
Abdelhafeez et al. [39]	2022	Moderate	Low	Low	Low	Moderate	Low	Low	Low
Altundag et al. [29]	2015	Low	Low	Moderate	Low	Moderate	Low	Low	Low
Birte-Antina et al. [40]	2017	Low	Low	Moderate	Low	Moderate	Low	Moderate	Moderate
Bratt et al. [41]	2020	Low	Low	Moderate	Low	Low	Low	Low	Low
Damm et al. [42]	2014	Low	Low	Low	Low	Low	Low	Low	Low
Denis et al. [43]	2021	Low	Low	Moderate	Serious	Moderate	Low	Low	Moderate
Fleiner et al. [44]	2012	Serious	Serious	Low	Moderate	Low	Low	Low	Serious
Geißler et al. [45]	2013	Moderate	Low	Low	Low	Low	Low	Low	Low
Gellrich et al. [46]	2017	Low	Low	Low	Low	Low	Low	Low	Low
Genetzaki et al. [47]	2021	Serious	Moderate	Low	Low	Low	Low	Low	Moderate
Gurbuz et al. [48]	2021	Moderate	Low	Low	Low	Low	Low	Low	Low
Haehner et al. [49]	2013	Low	Low	Low	Low	Low	Moderate	Low	Low
Hosseini et al. [50]	2020	Low	Low	Low	Low	Moderate	Low	Moderate	Low
Jiang et al. [51]	2019	Low	Low	Low	Low	Moderate	Low	Low	Low
Jiang et al. [24]	2017	Low	Low	Low	Low	Moderate	Low	Low	Low
Jiramongkolchai et al. [52]	2021	Moderate	Low	Low	Low	Low	Low	Low	Low
Knudsen et al. [53]	2015	Moderate	Low	Low	Low	Low	Low	Low	Low
Kollndorfer et al.[54]	2014	Moderate	Moderate	Low	Low	Low	Low	Low	Low
Konstantinidis et al. [55]	2016	Moderate	Low	Moderate	Serious	Low	Low	Low	Moderate
Konstantinidis et al. [26]	2013	Moderate	Low	Low	Moderate	Low	Low	Low	Low
Lamira et al. [56]	2019	Moderate	Low	Moderate	Serious	Critical	Low	Moderate	Serious
Langdon et al. [57]	2018	Low	Low	Low	Low	Low	Low	Low	Low
Le Bon et al. [58]	2021	Moderate	Moderate	Moderate	Moderate	Low	Low	Low	Moderate
Liu et al. [59]	2021	Moderate	Low	Low	Moderate	Low	Low	Moderate	Moderate
Mahmut et al. [60]	2021	Moderate	Low	Low	Low	Low	Low	Low	Low
Morquecho-Campos et al [62]	2019	Moderate	Low	Low	Low	Low	Low	Low	Low
Negoias et al. [63]	2016	Moderate	Low	Moderate	Moderate	Low	Low	Moderate	Moderate
Oleszkiewicz et al. [64]	2018	Low	Low	Low	Low	Low	Low	Low	Low
Oleszkiewicz et al. [23]	2022	Low	Low	Low	Low	Low	Low	Low	Low
Pellegrino et al. [27]	2019	Moderate	Low	Low	Moderate	Low	Low	Moderate	Moderate
Qiao et al. [65]	2019	Moderate	Low	Low	Low	Low	Low	Low	Low
Rezaeyan et al. [22]	2022	Low	Serious	Low	Low	Moderate	Low	Moderate	Moderate
Saatci et al. [25]	2020	Low	Low	Low	Low	Low	Low	Low	Low
Yan et al. [66]	2018	Moderate	Low	Low	Moderate	Low	Low	Moderate	Moderate
Yoon Choi et al. [67]	2021	Moderate	Low	Low	Low	Low	Low	Low	Low

### Pre vs post meta-analysis results

#### Olfactory function

Overall analysis of olfactory training on olfactory function shows pre–post meta-analytical effect of g = 0.755 (k = 45, SE = 0.093, CI 95% = [0.572, 0.937]). As heterogeneity within study effect sizes is statistically different from 0 (I2 = 86.64%, Q(44) = 286.545, p < 0.0001), moderator analysis was performed.

**Fig. 2:**
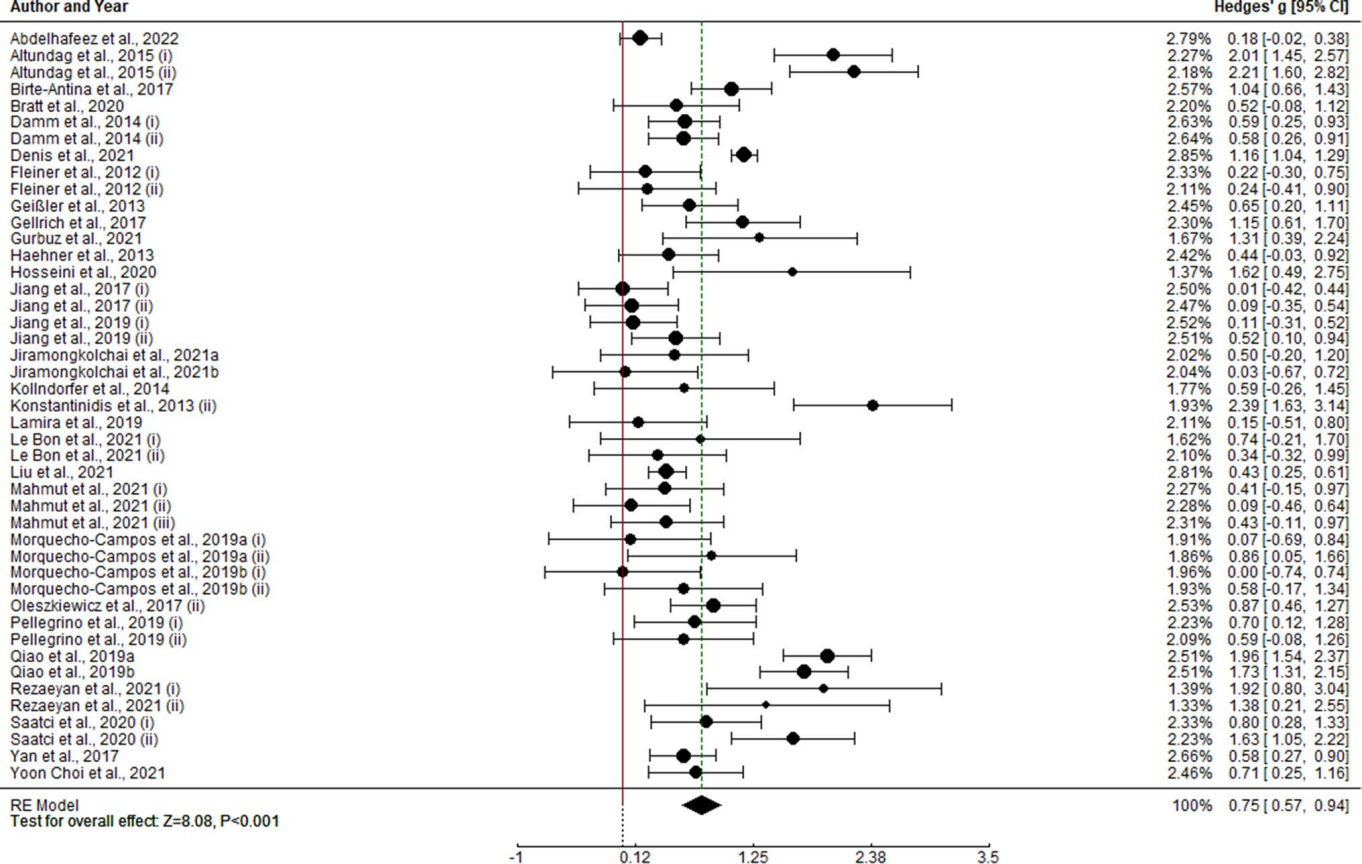
Forest plot for pre vs. post TDI score

Moderator analysis of potential predictors shows a statistically significant positive effect of duration of training period (k = 45, b = 0.027, Z = 2.145, p = 0.032) and age (k = 45, b = 0.025, Z = − 2.812, p = 0.005). Hence, longer training periods statistically predicts higher effect sizes and olfactory training effect tends to be higher in younger participants. No other moderator was able to account for model variability (p > 0.05). Regarding the model, heterogeneity accounted is R2 = 0.332 (Fig. 2).

#### Olfactory identification

**Fig. 3:**
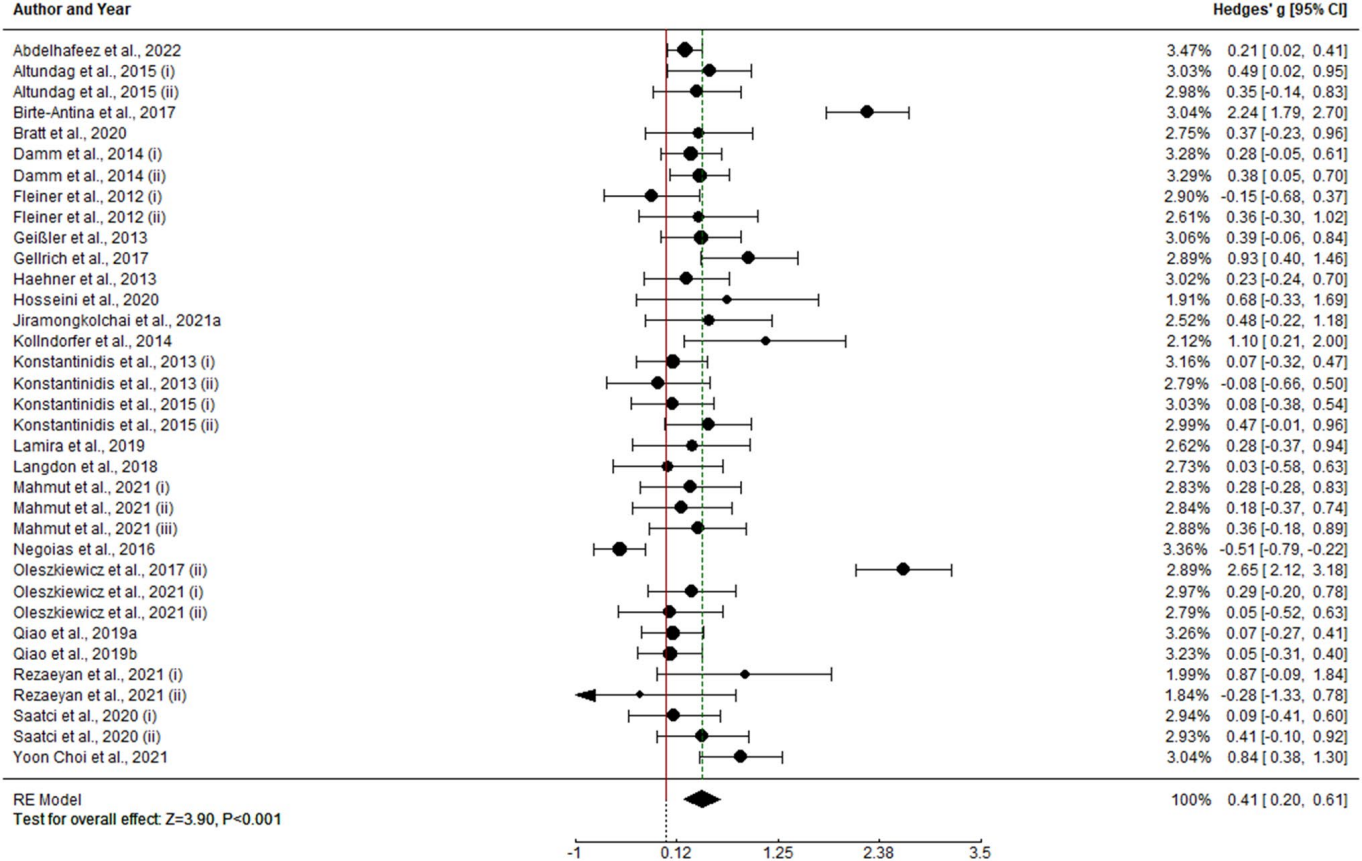
Forest plot for pre vs. post OI score

Analysis of olfactory training efficacy on olfactory identification performance shows pre–post meta-analytical effect of g = 0.843 (k = 35, SE = 0.139, CI 95% = [0.571, 1.114]). Heterogeneity percentage is similar to olfactory function, having I2 = 90.92% (Q(34) = 322.143, p < 0.0001). In this case, moderator analysis also points to duration of training (k = 24, b = 0.063, Z = 2.206, p = 0.027) and anosmia diagnosis (k = 24, b = 1.86, Z = 3.139, p = 0.0017) as predictors whose effects are positive and statistically significant. Therefore, regarding olfactory identification, longer training periods are related to higher effect sizes and, pre–post effect sizes tend to be higher in anosmic training groups. No other moderator was able to account for model variability (p > 0.05). Model with moderators accounts a R2 = 0.395 (Fig. 3).

#### Olfactory discrimination

**Fig. 4:**
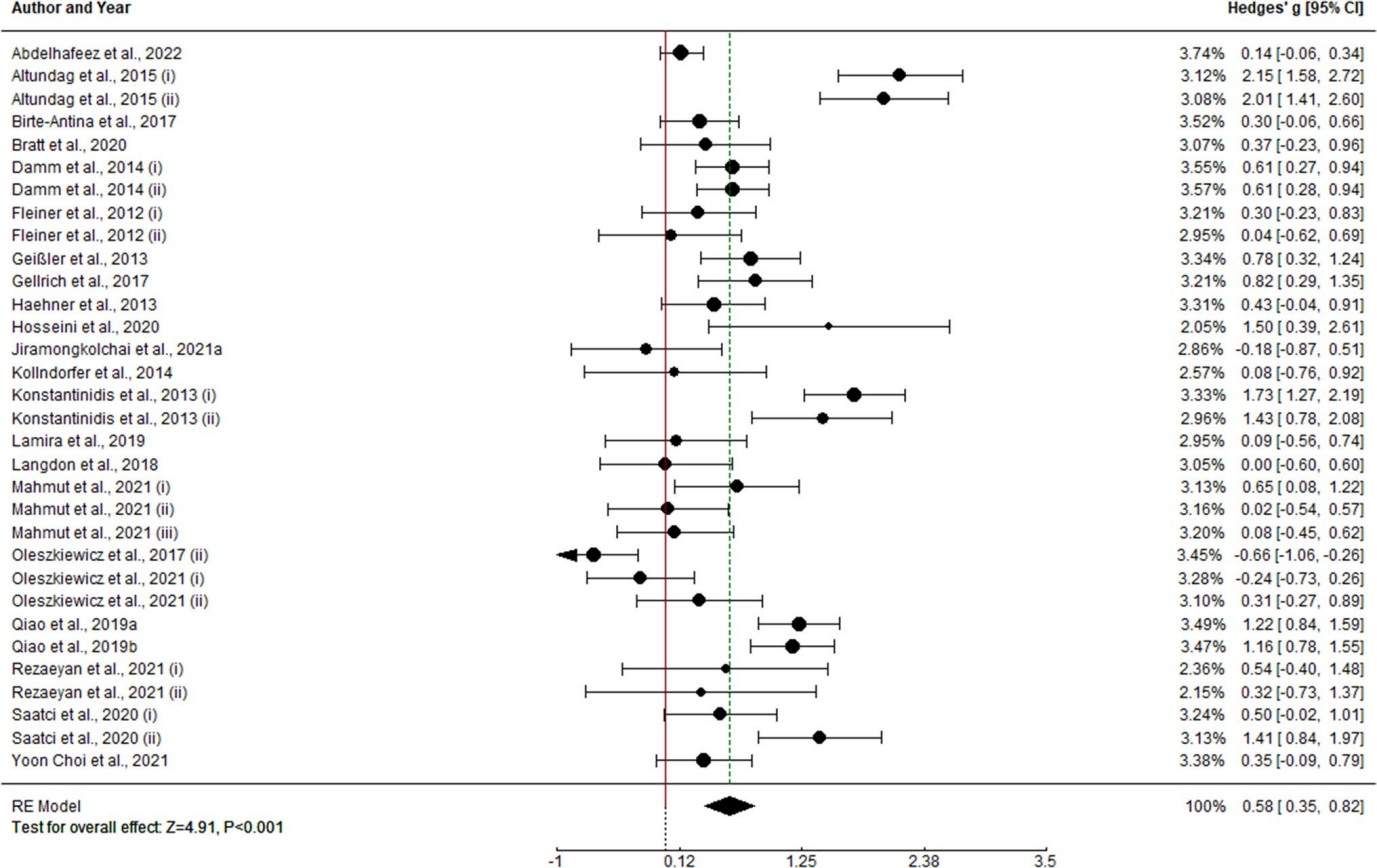
Forest plot for pre vs. post OD score

Pre–post meta-analytical effect of olfactory training on olfactory discrimination function is g = 0.585 (k = 32, SE = 0.119, CI 95% = [0.351, 0.818]). Heterogeneity within study effect sizes is also significant (I2 = 86.26%, Q(31) = 206.526, p < 0.0001). Therefore, moderator analysis of potential predictors was performed. In this case, other training (not classic nor personalized) statistically predicts lower effect sizes compared to only personalized training (k = 23, b = − 1.411, Z = − 2.586, p = 0.009). Besides, training tends to show higher effect sizes in olfactory discrimination in patients whose etiology is respiratory infections compared to other otorhinolaryngological issues (k = 23, b = 0.906, Z = 2.028, p = 0.042). No other predictor is statistically significant within the model (p > 0.05). Model with moderator’s accounts for R2 = 0.393 (Fig. 4).

#### Olfactory threshold

**Fig. 5:**
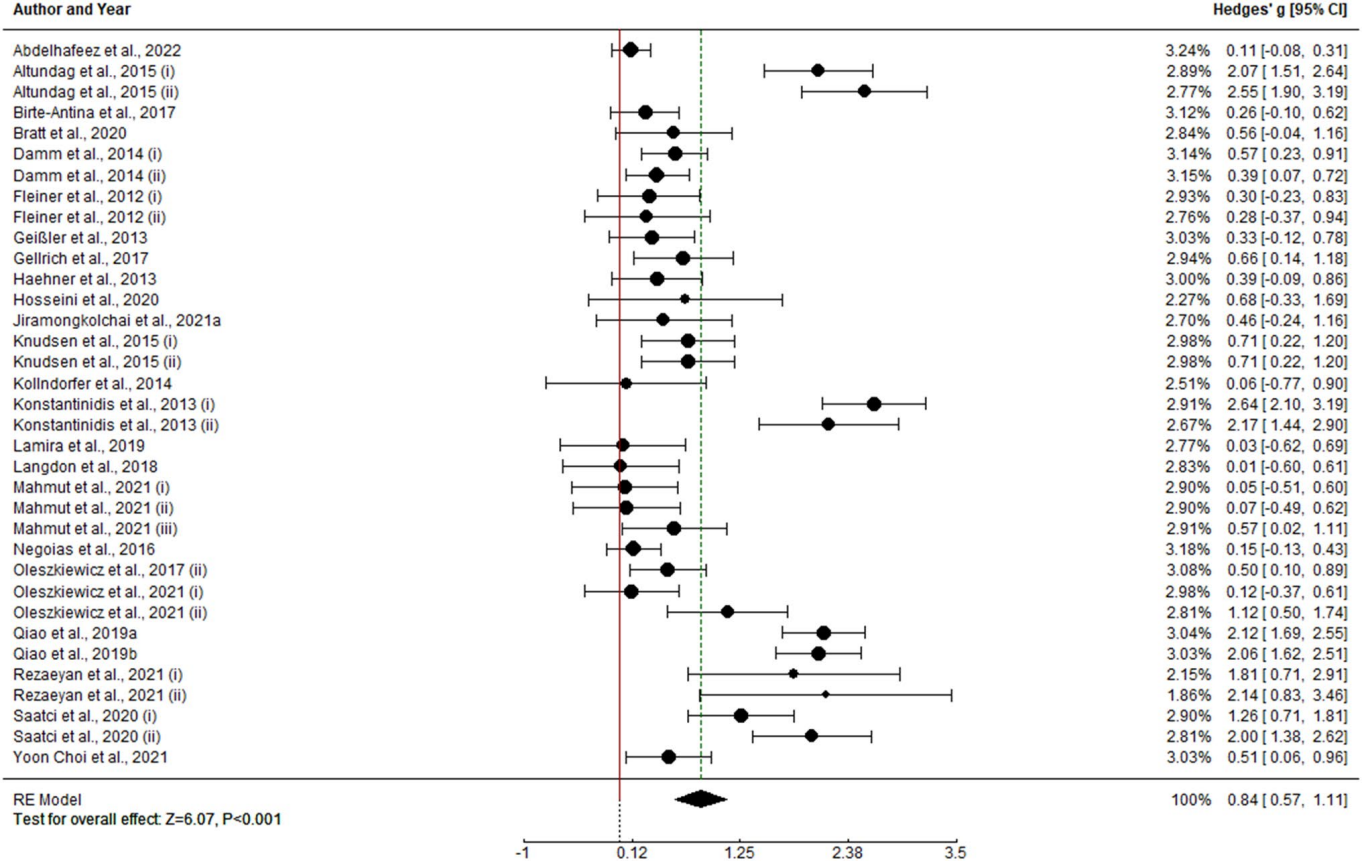
Forest plot for pre vs. post OT score

Pre–post effect sizes on olfactory threshold function resulted in a meta-analytic effect of g = 0.406 (k = 35, SE = 0.104, CI 95% = [0.202, 0.61]). As heterogeneity within study effect sizes is statistically different from 0 (I2 = 84.67%, Q(35) = 207.067, p < 0.0001), moderator analysis was performed, but no predictor was able to account for model variability (p > 0.05; Fig. 5).

### Experimental vs control meta-analysis results

#### Olfactory function

For the results of this section, meta-analysis was performed on experimental vs control effect sizes. Overall analysis of olfactory training on olfactory function shows experimental vs control meta-analytical effect of g = 2.124 (k = 8, SE = 0.363, CI 95% = [1.412, 2.836]). In this case, heterogeneity within study effect sizes is statistically different from 0 (I2 = 86.31%, Q(7) = 42.497, p < 0.0001). Therefore, moderator analysis was performed. Mean age shows a potential positive effect (k = 8, b = 0.036, Z = 2.393, p = 0.016). This effect could imply a tendency of olfactory training increasing its effect with age. However, this effect might be a statistical artifact, with only 8 primary results. By categorizing mean age moderator in [18–40), [40–60) and [60, > 60], we found just 2 studies with mean age below 40 years. Effect size in this group [18–40) is significantly lower within moderator analysis (b = − 1.96, Z = − 2.135, p = 0.033). Hence, this result may be explained by this statistical artifact. No other statistically significant effect was in any potential moderator variable (p > 0.05).

#### Olfactory identification

Analysis of olfactory training effectiveness on olfactory identification performance shows experimental vs control meta-analytical effect of g = 1.486 (k = 10, SE = 0.335, CI 95% = [0.829, 2.143]). Heterogeneity percentage is I2 = 89.37% (Q(9) = 87.248, p < 0.0001), so moderator analysis was performed. In this case, mean age (k = 9, b = 0.036, Z = 2.393, p = 0.016), duration of training period (k = 9, b = 0.053, Z = 2.565, p = 0.0103) and diagnosis of anosmia (k = 9, b = 0.894, Z = 2.056, p = 0.039) are suitable and statistically significant predictors of effect sizes. Hence, training effect size tends to be higher in older cohorts, also in samples with diagnosis of anosmia and with longer training periods.

#### Olfactory discrimination

Experimental vs control effect sizes on olfactory discrimination performance resulted in a meta-analytic effect of g = 1.088 (k = 9, SE = 0.219, CI 95% = [0.66, 1.517]). As heterogeneity within study effect sizes is statistically different from 0 (I2 = 74.99%, Q(35) = 31.502, p < 0.0001), moderator analysis was performed. However, no statistically significant predictor was found within the model (p > 0.05).

#### Olfactory threshold

**Fig. 6:**
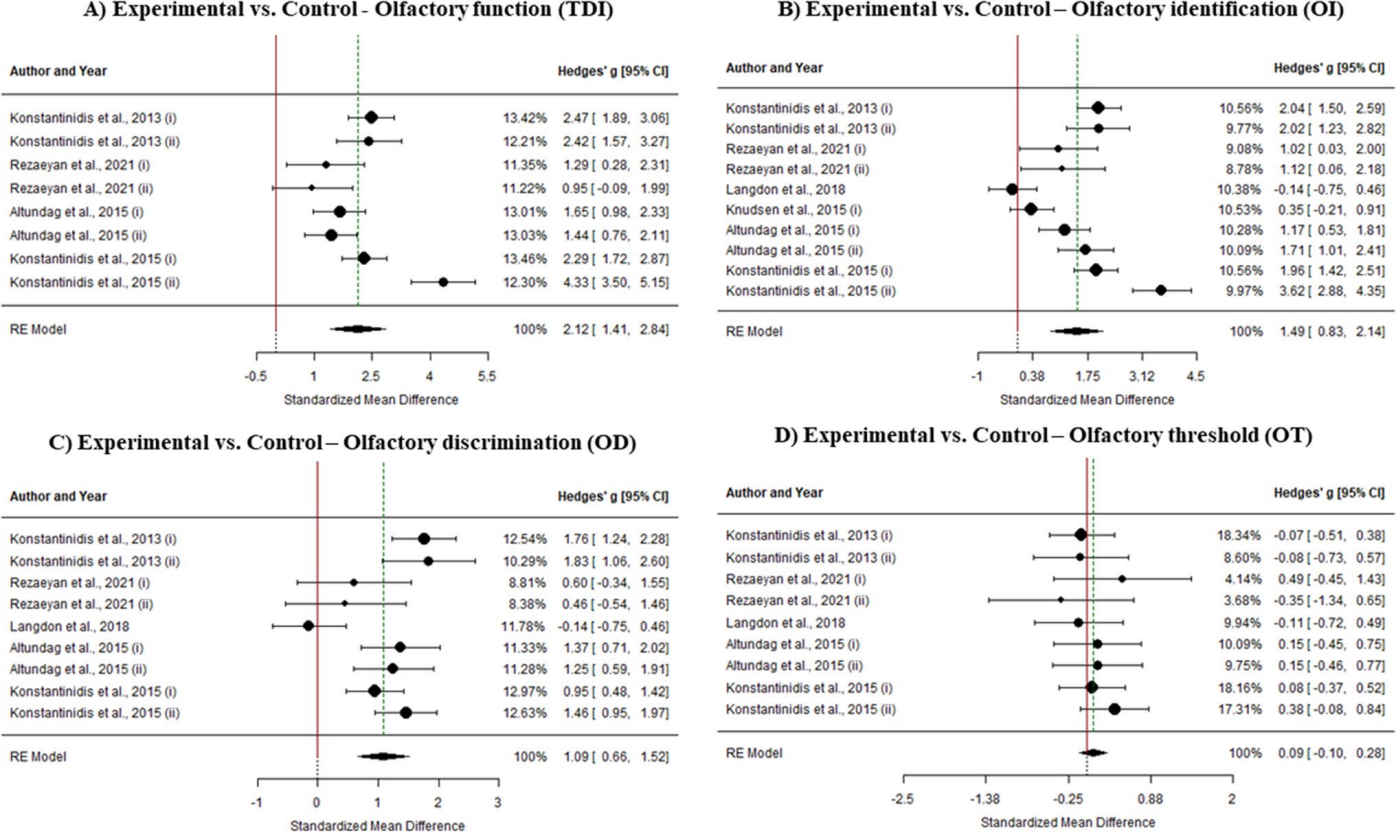
Forest plots for experimental vs. control analyses: **A** TDI score; **B** OI score; **C** OD score; **D** OT score

Meta-analysis of olfactory training effectiveness on olfactory threshold function points that there is no evidence to support an experimental vs control meta-analytical effect different from 0 (g = 0.087, k = 9, SE = 0.097, CI 95% = [− 0.103, 0.278]). No further analyses were performed in this case (Fig. 6).

### Pre vs follow-up meta-analysis results

#### Olfactory function

Finally, pre and follow up effect sizes were meta-analyzed. Overall analysis of olfactory training on olfactory function shows pre and follow up meta-analytical effects of g = 1.751 (k = 9, SE = 0.712, CI 95% = [0.352, 3.149]). Heterogeneity within study effect sizes is also statistically different from 0 (I2 = 98.02%, Q(8) = 211.262, p < 0.0001); so moderator analysis was performed. Anosmia diagnosis was found as a statistically significant predictor within the model (k = 6, b = 4.229, Z = 3.13, p = 0.0017), accounting for a R2 = 0.834.

#### Olfactory identification

Pre and follow up effect sizes on olfactory threshold function resulted in a meta-analytic effect of g = 1.355 (k = 10, SE = 0.421, CI 95% = [0.529, 2.18]). As heterogeneity within study effect sizes is statistically different from 0 (I2 = 94.63%, Q(9) = 137.627, p < 0.0001), moderator analysis was performed. Nevertheless, no predictor was found statistically different from 0 within the model (p > 0.05).

#### Olfactory discrimination

Analysis of olfactory training effectiveness on olfactory threshold function shows experimental vs control meta-analytical effect of g = 1.498 (k = 8, SE = 0.54, CI 95% = [0.442, 2.554]). Heterogeneity percentage is similar to olfactory function, having I2 = 95.41% (Q(7) = 129.536, p < 0.0001). However, moderator analysis points to no variable as a statistically significant predictor within the model (p > 0.05).

#### Olfactory threshold

**Fig. 7:**
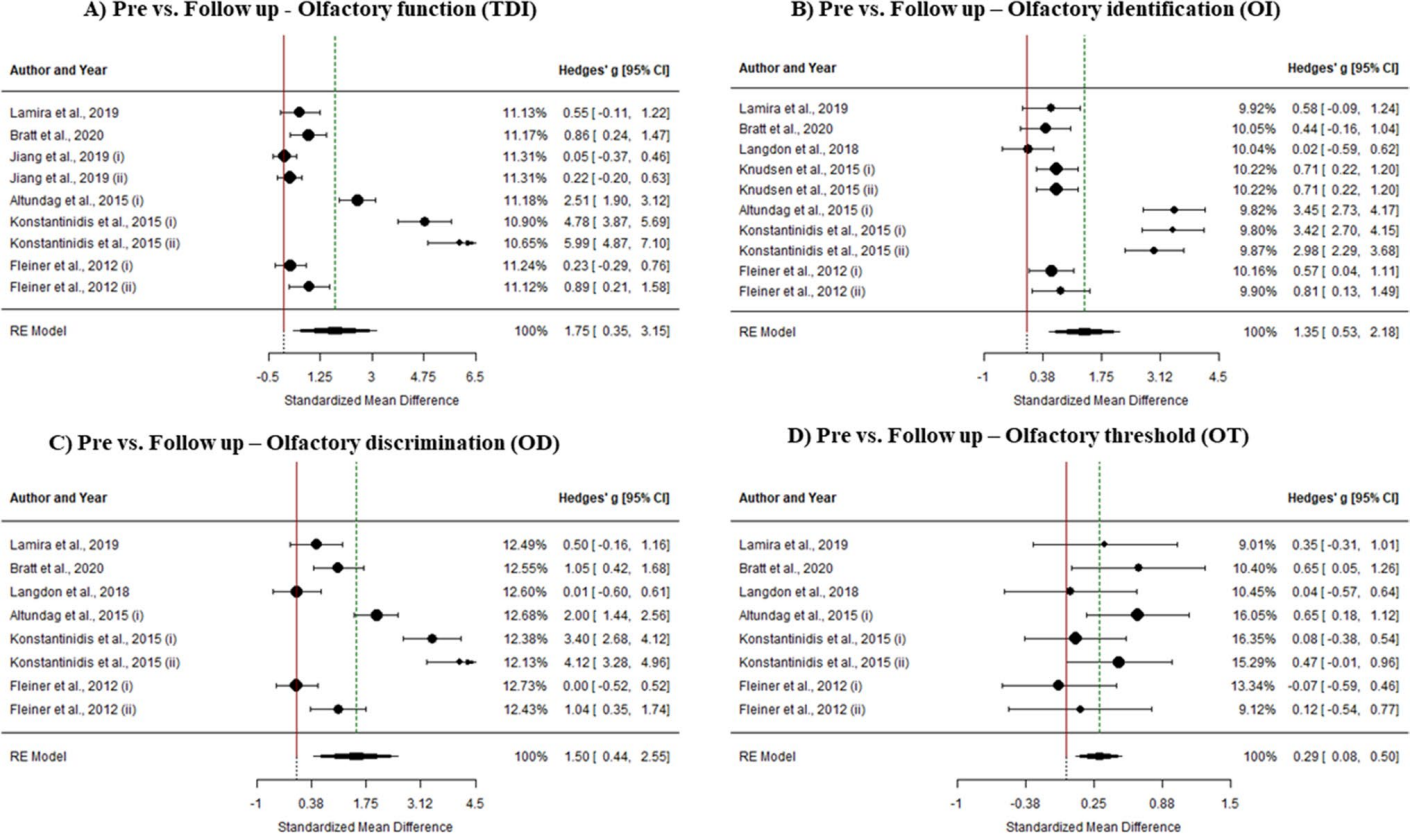
Forest plots for pre vs. follow-up analyses: **A** TDI score; **B** OI score; **C** OD score; **D** OT score

Finally, pre and follow up effect sizes on olfactory threshold function resulted in a meta-analytic effect of g = 0.295 (k = 8, SE = 0.102, CI 95% = [0.084, 0.504]). Analysis of heterogeneity within study effect sizes gives an I2 = 15.99%, which gives no evidence of being statistically different from 0 (Q(7) = 7.767, p = 0.353). Therefore, no moderator analysis was performed (Fig. 7).

#### Publication bias

Analyses of publication bias were performed over pre and post meta-analysis models (TDI, Olfactory Identification, Olfactory Discrimination and Olfactory Threshold scores), as those ones comprise the complete sample of studies. Experimental vs. Control and Pre and Follow up meta-analyses were estimated with subsamples (not every study had control group or follow-up measurement). Having this in mind, several analyses were performed in order to account for publication bias.

**Fig. 8:**
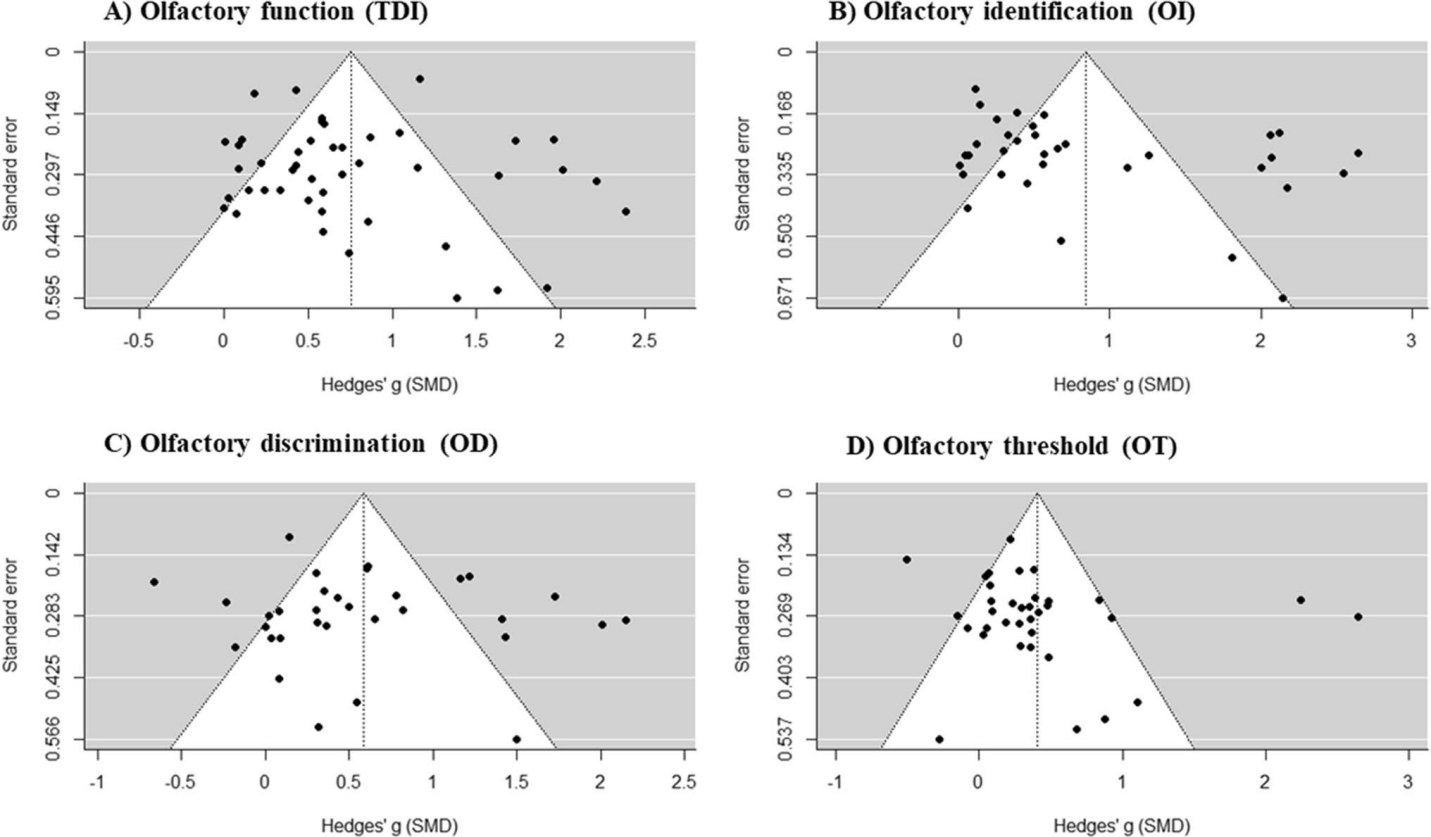
Funnel plots for publication bias in pre vs. post analyses: **A** TDI score; **B** OI score; **C** OD score; **D** OT score

Firstly, funnel plots for pre–post effects are shown in Fig. 8. Visual inspection of the funnel plot for olfactory function (A) shows a slight left asymmetry, which may imply an underestimation of meta-analytic effect size. However, Egger’s test does not allow interpreting this potential asymmetry as statistically significant (Z = 1.303, p = 0.193). Following, Duval and Tweedie’s trim and fill method was applied to this model. This procedure points that no significant number of studies were missing from analysis, as a very similar effect size was estimated (g = 0.756). To conclude, fail-safe N was calculated through Rosenthal [37] procedure and 6912 null studies would be required to cancel the observed effect.

Regarding olfactory identification model, funnel plot (B) also points to visual asymmetry, with few studies showing effects sizes higher than g = 2 and other studies with effect sizes closer to 0. In this case, Egger’s test also does not allow interpreting this potential asymmetry as statistically significant (Z = 1.886, p = 0.059). Besides, trim and fill method was applied, giving an estimated effect of g = 0.843, pretty close to the original one. Hence, no missing studies might confound the estimated effect size. Fail-safe N indicates that 4007 null studies would be necessary to include in the analysis to elude the estimated effect size.

Preceding, olfactory discrimination funnel plot (C) is presented, which shows no visual evidence of asymmetry. Egger’s test is consistent with it, as there is no evidence of potential bias in the funnel plot (Z = 0.34, p = 0.734). Duval and Tweedie’s trim and fill analysis agrees with this line, as no missing studies are disturbing the analysis (trim and fill effect size of g = 0.585). Finally, fail-safe N equals to 1823 null studies in order to cancel the estimated effect size.

Finally, potential publication bias was analyzed on the olfactory threshold model. Its funnel plot (D) is also slightly asymmetrical on the left, but Egger’s test gives no evidence of an underestimation of effect size (Z = 0.855, p = 0.392). Trim and fill analysis reveals that no missing studies are noising the effect size estimation (g = 0.406) and fail-safe N points that 1015 null studies should be added to the model in order to fade the effect.

To conclude, these results support the statement that the current sample of primary studies might be a representative sample of all the studies about the efficacy of smell training in olfactory dysfunction.

## Discussion

Olfactory dysfunction affects different categories of quality of life and daily life activities (ADLs), such as food enjoyment, personal hygiene, vital personal security, and it reduces the retrieval of memories associated with olfaction [1, 3, 68]. Emphasizing the hazardous effects that lead to inability to sense noxious chemicals, smoke, gas and spoiled food [69, 70] presents a great threat to one’s safety. Considering the importance of the sense of smell, studies have investigated its link with cognition and emotional status, they have observed olfactory deterioration in patients with neurodegenerative diseases [43, 44, 71]. Previous studies have reported that 25% to 33% of patients with olfactory dysfunction have symptoms of depression, and 27% to 30% indicate severe distress on general quality of life questionnaires due to their reduced sense of smell [3]. Further, physiologic anorexia, common in geriatric populations, may be partly due to diminished olfaction [3, 72].

Olfactory dysfunction is a problem with a variety of proposed etiologies, with postinfectious, posttraumatic, chronic inflammatory (rhinosinusitis, rhinitis), neurodegeneration causes being among the most common [72–77].

Olfactory loss is a challenging clinical problem with few proven therapeutic options. A wide range of treatment modalities for anosmia and hyposmia including corticosteroids [58], theophylline [78], antibiotics [79], and acupuncture have been attempted [79]. Non-pharmacological and non-invasive approaches have also been studied, such as olfactory training as a therapeutic approach [29, 80].

In the presented meta-analysis, it was analyzed previous smell training studies to provide a quantitative estimate of the effectiveness of olfactory training across four different olfactory domains—smell identification, discrimination, threshold and olfactory function (TDI), as well as specifying the potential moderator variables that influence olfactory recovery.

The primary objective of present meta-analysis was to evaluate the effectiveness of olfactory training as a therapeutic intervention for the rehabilitation of olfactory dysfunction. This study’s analyses demonstrate a positive and statistically significant effect of olfactory training in all olfactory abilities: with large effects of training on identification (g = 0.843), discrimination (g = 0.585) and TDI-score (g = 0.755), and small-to-moderate effect of threshold for odor detection (g = 0.406). When comparing pre–post performance in experimental groups (those who were submitted to the OT process due their OD). Those statistically significant effects were also observed when comparing olfactory capacity post olfactory training in experimental and control groups identification (g = 1.486), discrimination (g = 1.088) threshold (g = 0.087) and TDI-score (g = 2.124). And maintained when comparisons were drawn between pre and follow-up analysis identification (g = 1.355), discrimination (g = 1.498) threshold (g = 0.295) and TDI-score (g = 1.751). This overall effectiveness of the olfactory training is encouraging, given the importance of olfaction and inconsistent results regarding other forms of olfactory dysfunction treatment.

When comparing the experimental and control group there was no significant evidence to support OT effectiveness. In addressing the limited improvement in olfactory threshold observed following OT, it’s essential to assess the training protocol’s specific components. It is known that OT training protocols are more focused on discrimination and identification tasks, rather than targeting olfactory threshold directly, this could explain the modest improvements. Future studies should explore tailored training techniques explicitly designed to enhance olfactory threshold, such as incorporating exercises that challenge participants’ ability to detect minimal scent concentrations.

The second objective of this meta-analysis was to investigate the relationship between the observed olfactory training outcomes, duration of the training period, participants characteristics such as age, etiology and type of OD and OT method.

Interestingly, when comparing the results between experimental and control groups, in olfactory function (TDI), age presented a significant positive effect (b = 0.036, Z = 2.393, p = 0.016), although being known to having a negative impact on olfactory function [80–85]. This could be attributed to a statistical artifact. We categorized the mean age moderator in [18–40), [40–60) and [60, > 60] cohorts, with just 2 studies with mean age below 40 years. Meta-analytical effect size in these 2 studies is significantly lower compared with the other age groups and age cohorts are too reduced in sample size of studies to establish a conclusion. Age is widely recognized as a significant factor influencing olfactory function, with a notable decline typically observed around the age of 60 [64, 80, 83], Interestingly, the majority of studies included in this meta-analysis featured samples composed of participants aged 40 years or older [42, 46, 48]. This trend may be attributed to two primary factors. Firstly, younger participants are often associated with higher levels of neural plasticity, rendering them more susceptible to spontaneous improvements in olfactory function [11]. Secondly, volunteer-based investigations, which commonly comprise the participant pool in research studies, may inherently skew towards older age groups due to various biases [86].

Anosmia was considered a moderator variable due to its potential impact on the effectiveness of olfactory training interventions. Patients with anosmia typically exhibit a more pronounced olfactory dysfunction at baseline, reflecting the severity of their condition. Consequently, they often have a larger scope for improvement in olfactory function following interventions like olfactory training. This is because the extent of improvement can be more noticeable and impactful in individuals with anosmia, who start from a lower baseline level of olfactory function. Consequently, anosmia was considerate as a moderator in this meta-analysis since it allows researchers to explore how the severity of olfactory deficits influences the response to olfactory training interventions.

Statistical analysis also pointed out that “training duration” and “anosmia diagnosis” are predictors, whose effect on the outcome of olfactory training, was found to be positive and significant in olfactory identification on both pre–post and experimental-control analysis.

The same result regarding “duration of training” was observed on the meta-analysis of Sorokowska et al. [87], as well as in other studies [45, 55]. Nonetheless, it is noteworthy to specify that there is a probable ceiling effect after a certain training period, the potential ceiling effect of olfactory training presents an important consideration in its efficacy. Olfactory training involves repetitive exposure to odorants to stimulate olfactory neural pathways and improve olfactory function [88–90]. However, there may be a limit to the extent of improvement achievable through this method. Over time, individuals may reach a plateau where further gains in olfactory function become increasingly challenging to attain. This ceiling effect could be attributed to various factors, including the severity of olfactory dysfunction, the duration and intensity of training, and individual differences in neural plasticity [20, 45]. To address this, it's crucial to tailor olfactory training protocols to individual needs, considering factors such as baseline olfactory function and response to training. Additionally, determining the optimal duration for olfactory training is essential. Regarding OT, the duration of the training period remains a topic of ongoing debate [25, 27, 29]. The evidence presented in the literature showcases a range of perspectives, highlighting both the potential benefits of longer training periods and the practical considerations surrounding the optimal duration. It has been observed an improvement in olfactory function of 11% to 68% after 12–16 weeks of OT [26, 44].

However, Konstantinidis et al. [55] compared patients who performed OT for 16 and 56 weeks and found that the prolonged training benefited the proportion of subjects exhibiting clinically significant improvement (58% vs 71%, respectively). Nevertheless, the same study by Konstantinidis provides valuable insights into the stability of OT effects over time. For it showed that the effects of OT are stable over time and the improved olfactory performance of patients who underwent 16 weeks-long OT remained unchanged for the next 40 weeks, leading to the belief that OT should be extended to a minimum of training period of 16 weeks.

Regarding the “anosmia diagnosis” positive relationship with OT, it was seen in previous studies that anosmic patients, for being those who naturally had more to benefit from OT, presented better results after the training period, increasing their overall olfactory capacity [27, 91, 92].

The analysis revealed that the type of treatment administered did not yield statistically significant differences in outcomes. Whether studies exclusively utilized olfactory training or combined it with medication, the results did not demonstrate a substantial variation. It’s important to consider that some patients may have been receiving medications unrelated to olfactory loss (e.g. for hypertension or anti-Parkinson’s treatment). This introduces a potential confounding factor that could influence the outcomes of the analysis. Despite the presence of these medications, the results still did not indicate a statistically significant difference based on the type of treatment administered. This suggests that even in the presence of medications targeting other conditions, the combined approach of olfactory training with additional medications did not show a clear advantage over olfactory training alone in improving olfactory function.

When drawing comparisons between experimental and control groups the meta-analytical effect was close to 0 (g = 0.087], meaning that there is no statistical difference between both groups. A statistically significant difference was only found in the Kolldorfer and colleagues study [54], and such significance might be due the small sample size.

The same comparison between experimental and control groups, also pointed out that patients with OD due to post-viral or respiratory etiology gained more from OT, which was also observed in different studies [45, 52, 71, 87].

This meta-analysis revealed a substantial level of heterogeneity exceeding 80% in all conducted analyses. This heterogeneity could potentially be attributed to the limited sample sizes of the participants in the included studies. Furthermore, the overall quality assessment of the included studies indicated a moderate risk of bias, consistent with other meta-analyses investigating olfactory training [87, 93]. These findings emphasize the importance of addressing methodological limitations and enhancing the rigor of future studies in this field.

Another limitation that should be considered in future studies is the inclusion of disease duration, it's crucial to acknowledge that the inclusion of disease duration represents an important factor that could significantly impact the results. Therefore, it should be treated as a potential moderator and explored in future research.

The current study suggests that olfactory training might be a therapeutic modality for olfactory dysfunction. The majority of the studies reported positive outcomes in olfactory function without those possible side effects that can be seen if pharmacological or surgical interventions [90, 93]. This meta-analysis was consistent with the reported results on individual studies [26, 55, 92] and on systematic reviews and meta-analysis [87, 88] published with revealing improvements on olfactory function after training.

## Conclusion

This study reveals a notable and statistically significant positive impact of olfactory training on the enhancement of all domains of olfactory function. Olfactory dysfunction represents a recurring challenge encountered across diverse etiologies. Although the precise mechanism by which olfactory training effectively restores or improves olfactory function remains elusive, this comprehensive meta-analysis provides compelling evidence that justifies further exploration of this therapeutic modality. Moreover, considering its proven efficacy, cost-effectiveness, and non-invasive nature, olfactory training merits careful consideration and integration into clinical practice as an efficacious and safe intervention.

## Data Availability

This is a meta-analysis of published work. All data are in the public domain in the form of manuscripts. Any and all data that we report are presented in either the body of the manuscript or the online supplemental material.
